# Research Progress of Wood Cell Wall Modification and Functional Improvement: A Review

**DOI:** 10.3390/ma15041598

**Published:** 2022-02-21

**Authors:** Ting Zhou, Honghai Liu

**Affiliations:** 1 College of Furnishings and Industrial Design, Nanjing Forestry University, Nanjing 210037, China; muyit@njfu.edu.cn; 2 Jiangsu Co-Innovation Center of Efficient Processing and Utilization of Forest Resources, Nanjing Forestry University, Nanjing 210037, China

**Keywords:** wood, cell wall, modification, functional improvement, nanotechnology

## Abstract

The modification of wood cell walls is based on the characteristics of the chemical composition and structure of the cell wall. Various physical and chemical modifications to these characteristics enhance the original properties of the cell wall and give additional functionality. Through complex modification, wood has also obtained the opportunity to become a multifunctional material. Scholars have paid more attention to the microscopic properties of the cell wall with continuous enrichment of modification methods and improvement of modification mechanisms. This article summarizes the methods of cell wall modification in recent years and proposes prospects for future development: (1) innovation of modifiers and combination with modification mechanism, as well as improvement of cell wall permeability; (2) the application directions of cell wall structures; and (3) the application of nano-technologies in cell wall modification. This review provides further ideas and technologies for wood modifications.

## 1. Introduction

Wood is a renewable and biological material, which is widely used in various fields [1,2,3,4], ranging from industry, energy, and aerospace to products of furniture, tableware, and daily necessities. As a biological material, wood is widely used in many fields but also has certain limitations in nature, such as poor dimensional stability, biological durability, and vulnerability to microorganisms and pests, therefore innovative wood drying [5] and modification [6] technologies have emerged to improve the properties to satisfy various utilizations demand. In recent decades, wood modification technology has developed steadily. The defects of natural wood are removed or repaired after modification and most of the disadvantages are improved. In the review that has been published, Spear’s review of wood modification comprehensively summarized the latest progress in wood functional modification, and they pointed out the feasibility of wood nanotechnology in her conclusion [6]. In terms of microstructure, cells are the basic units of wood composition [7], the cell wall of wood constitutes the main form of wood, and thus the structures of the cell wall determine the properties of wood products [8]. Essentially, wood modification is the study of wood cell wall modification at the cellular level.

Wood cell walls are composed of cellulose, hemicellulose, and lignin. Celluloses construct the skeletons of the cell wall, and the space between the skeletons is occupied by the hemicelluloses and by lignin [9]. The cell walls consist of a complex combination of these three substances, which give basic physical and chemical properties. At the same time, the fiber of wood determines its anisotropy. Wood fibers have different physical properties in chordwise, radial, and longitudinal directions (growth direction), (e.g., the difference between chordwise drying shrinkage rate and radial drying shrinkage rate, the same behavior in the longitudinal direction). Meanwhile, the anatomical characteristics of the wood structure have different advantages and disadvantages in different species and parts of the wood.

In wood modification, the cell walls, as a carrier, not only reacts with the added modifiers but also fix the modifier using its structure to produce a physical combination with the reagent to enhance performance. For the modification of wood cell walls, the particularly important points are the moisture absorption and permeability of wood cell walls. A lot of studies have proved that moisture can change the properties of wood, resulting in great shrink and swell of wood products [10]. Therefore, many modifications are dedicated to reducing moisture absorption properties of wood and obtaining better dimensional stability. However, a single characteristic improvement cannot meet the utilization demand of wood, thus multi-functionality modifications of wood were developed to meet further demand. The water-resistance and dimensional stability of lumber have certain defects (in natural conditions, the moisture content of timber changes, to a small extent), and low resistance to biotic factors (insect, microorganism) and abiotic factors (illumination, temperature), flammability. Therefore, more chemical modifiers are used to stabilize and enhance the performance of wood. When chemical reagents are added, the permeability of the cell wall is particularly important. The micro-pores in the cell wall are the channels and reaction site of the reagent, which determine whether the modifiers can enter the cell cavities and complete the filling of cell walls. The penetration process of the modifier in the cell wall is of great significance for the study of wood modification [11].

This article summarizes the methods of wood cell wall modification and the application of modified materials in recent years and proposes a prospect for the development of wood cell wall modification in the future.

## 2. Thermal Modification on Wood Cell Walls

Thermal modification (TM) of wood enhances wood performance by reducing moisture absorption, improving dimensional stability, and biological durability [12,13,14]. Wood TM can be conducted under air, vacuum, steam, or other inert gases [15]. The TM temperature is usually between 160 °C and 260 °C. But they are different in terms of treatment conditions, such as the conditions with protective gases (such as nitrogen or steam), wet or dry, the use of oil, etc. [16]. When the temperature is between 20 °C and 150 °C, the wood undergoes the process of drying, in which, firstly free water is lost and, finally, bound water. As the temperature rises from 180 °C to 250 °C, the wood undergoes a TM process. Wood undergoes great chemical transformations, and its physical and chemical properties change. When the temperature exceeds 250 °C, the wood begins to undergo carbonization and pyrolysis, generating carbon dioxide and other degradation products [16].

TM enhances the function of wood by changing the composition of wood cell walls. The chemical composition changes through the degradation of cell wall materials and extracts at high temperatures. The degradation of cell walls materials [17] leads to effects on wood properties [18]. The change in composition results in a decrease in wood density and pH. The change of chemical composition caused by TM depends on the duration, pH, and temperature, and temperature is the most significant factor [19].

### 2.1. Steam Thermal Modification

Wood in steam TM, the steam can not only prevent the wood from burning but also participates in a certain chemical reaction. Tian et al. improved the bonding performance of Masson pine using steam TM. Although the surface of wood cell walls undergoes a series of changes, such as collapse, shrink, deformation, etc., wood performance (surface wettability, hydrophobicity and bonding performance, etc.) improved. Masson pine wood treated at the temperature between 160 °C to 180 °C has a higher surface wettability, while the wood obtained good hydrophobicity when treated at 200 °C to 220 °C. The reason for this phenomenon is that the surface energy of wood cell walls decreases with an increase in temperature and the loss of hydroxyl groups during TM [20]. Cao et al. treated the Chinese fir under steam protection (protect the wood from oxygen), and pointed out steam TM significantly improved the dimensional stability of the Chinese fir [21]. Elin et al. combined the technology of wood surface densification and superheated steam TM and proved that this combination can improve the mechanical properties and dimensional stability of low-density wood. The micromechanical properties of dense wood on the surface have been enhanced, and the superheated steam treatment further improved the crystallinity and crystal size of the wood cell wall. These enhance the properties of the wood from the microstructures [22]. The steam TM has long been considered an effective means to improve the dimensional stability, durability, and decay resistance of wood, but in the complex chemical modification, whether water vapor participates in the reaction needs to be focused on.

Other thermal modifications use gases as heat transfer media, such as nitrogen, which rarely reacts with wood. Bytner et al. thermally modified Populus Nigra L. in a nitrogen atmosphere to improve its dimensional stability. He also believes that higher modification temperatures and longer processing times contributed to a lower swelling anisotropy [23].

On the other hand, liquid can also be used as a heat conduction medium in wood thermal modification. In the study of oil heat treatment, Wang et al. found that the results of his study confirmed that chemical reactions in the wood resulting from the heat treatment account for the main improvements of wood properties in reduced hygroscopicity and improved dimensional stability, while the oil absorbed by wood reduces the rate of water absorption [24].

### 2.2. Vacuum Thermal Modification after Glycerin Pretreatment

The vacuum environment can effectively prevent the wood from burning during the TM process. Sivrikaya et al. impregnated wood under vacuum condition (0.08 MPa) for 1 h with dissolved glycerin before vacuum TM [25]. The advantage of vacuum impregnation to wood is that the intervention of glycerin promotes the degradation of cell wall materials, and the TM can be carried out at a lower temperature. Glycerol et al. also provide additional hydroxyl groups in the cell wall, forming a three-dimensional network and stabilizing the wood cell wall polymers [26]. This TM enhances the bending properties of wood and significantly improves the flexibility of wood cell walls [25].

### 2.3. Impregnation Combined with Thermal Modification

The chemical composition of wood cells contains a large number of hydroxyl groups, especially the polysaccharides, leading to dimensional instability of wood material [27]. In order to obtain better dimensional stability, various physical and chemical methods are used to change the original properties of wood. The use of polyethylene glycol (PEG) to fill wood cell walls can achieve good dimensional stability, but PEG also has some disadvantages, such as high cost and non-green. In addition, phenolic and urea-formaldehyde resins are also used for modification, which is not ideal in practical applications due to the production of toxic substances. Therefore, renewable non-toxic modifiers have lately received the most attention. Zhang et al. used suberin monomers (SMs) to TM wood to improve the dimensional stability. They firstly impregnated poplar wood with SMs and then conducted a TM. The swelling of TM wood is significantly reduced, leading to an obvious improvement in dimensional stability. The reason may be attributed to SMs filling in the wood cell cavities during immersion, which blocks the internal migration of water and also reduces the contact between the cell walls and moisture. Meanwhile, SMs penetrate into wood cell walls and expand over there, filling in pores and cavities of cell walls, thus reducing swelling of cell walls [28].

TM improves wood dimensional stability and decay resistance, but darkens wood color [29]. The surfaces of TM wood undergo severe photochemical degradation as exposed to the outdoors [30]. Therefore, further combined modifications are developed to improve the weather resistance of TM wood [31]. Shen et al. (2018) used TiO_2_ sol and paraffin emulsion to impregnate the wood, and then TM the samples (*Pinus sylvestris* L. sapwood) at a high temperature. The anti-weathering performance of TMW was greatly improved by applying a combined post-treatment of TiO_2_ sol and paraffin emulsion resulting from simultaneous control of ultraviolet and water [32]. Lin used a PEG solution to impregnate rubber wood (*Hevea brasiliensis*) and then modified the wood thermally. The results show that PEG is physically adsorbed on the walls and pits of the wood, and there is no new chemical reaction between PEG and wood [33]. Peg penetrates into the interior of the wood cell wall and makes it expand, which reduces the water absorption and dimensional stability of wood [34]. These modifications for pores and cavities of cell walls require that the diameter of modifiers is smaller than that. After the modifiers penetrate the cell wall, thermal or other treatment are applied to induce reactions inside the cell wall, which partially isolates water from contact with the wood cell wall.

### 2.4. Thermo-Hydro and Thermo-Hydro-Mechanical Process

Thermo-hydro and thermo-hydro-mechanical processes, in an orthodox definition, no additives are used in the processes beyond water in combination with wood, heat, and external forces to shape the wood [35]. Procedures including impregnation or gluing to lock a shape are, however, usually included in these modification processes. Andersone et al. studied the chemical changes of birch wood occurring at thermo-hydro-treatment at temperatures of 150, 160, and 170 centigrade. The holocellulose and hemicellulose contents decrease with increasing thermo-hydro-treatment temperature.

The thermo-hydro-mechanical (THM) process has a positive effect on low-density wood, the elastic modulus and rupture modulus of dense wood increased, and the hardness of dense wood was also increased compared to normal wood [36]. Michelle et al. held the samples (fiber-grown *E. nitens*, saw-log managed *P. radiata*, and native eucalypt species from Tasmania) at 170 °C for 3 min and then heated them at a pressure of 300 kN, finally increasing the basic density of the three samples. The results showed that there were obvious differences among different varieties, and the application of fiber-grown *E. nitens* also showed good wood properties [37]. And Wang et al. treated the poplar wood by combining bulk densification and heat treatment, they found the best preheating temperature and time for the bulk densification process were 180 °C and 361 s, respectively. And the optimal heat treatment temperature and time were 190 °C and 3 h, respectively. The treated wood has the highest storage modulus and the lowest loss factor than other conditions [38].

### 2.5. Laser Surface Thermal Modification

Laser TM to the wood surface is different from traditional TM. A laser as a heat source is used to radiate wood surface, different components of wood materials (especially extracts and lignin change their structures during the heat-induced discoloration causes the wood to change) color; the plasticization of lignin and the increase of crystallinity of cellulose reduced the wettability of wood surface; the decrease of nutrient content such as sugar and protein in wood can improve the corrosion resistance of wood [39]. Fukuta et al. studied and concluded the best wavelength for wood processing. For CO_2_ laser modification, although the absorption coefficient of wood components is different, 355 nm is the best wavelength for modification [40]. Laser TM changes color and improves hydrophobicity of wood surface, the surface of the wood is carbonized, the roughness increases significantly, and enhances decay resistance of the wood [41]. Laser TM may be able to purposefully modify wood cell walls more precisely from a microscopic point of view and could be introduced into other methods to conduct more precise modification.

## 3. Chemical Modification on Wood Cell Walls

In the chemical modification of wood, modifiers are mainly used to affect the process of hydrolysis, oxidation, reduction, dehydration, or reaction with free radicals to improve the functionality of wood [42]. Improving the interaction between modifiers and wood cell walls are also critical for wood modification [43]. Meanwhile, the cell walls in acidic or alkaline environments exhibit different properties. The cellulose crystallinity index and crystallinity of wood treated in alkaline conditions increase, thus resulting in an increase in the elastic modulus of the cell wall [44]. The composition and physical properties of the cell wall dominate the properties of wood, thus chemical modification is mainly aimed at the material of wood cell walls. The development of chemical modification in recent years has focused on non-toxic and environmentally friendly reagents.

### 3.1. Acetylation

Acetylation modification technology is an earlier modification method [11], which can reduce the equilibrium moisture content (EMC) of wood, increase dimensional stability, weather, and decay resistance of wood [42]. The mechanism could be explained from two aspects, one is swelling the wood cell wall to the elastic limit, and another is replacing the hydroxyl groups of the cell wall material with a weakly hydrophilic group. Digaitis et al. achieved the targeted treatment of acetylation in their recent study. Through the control of reaction conditions, the interface of the cell wall and lumen is acetylated alone, or the entire cell wall is uniformly modified to varying degrees. This study is of significance to the distribution of moisture in wood, decay resistance, and dimensional stability [45]. Although acetylation can modify wood by reducing the number of hydroxyl groups on wood cell walls, the acetylated wood is deacetylated during the brown rot process, thus Thygesen et al. (2021) used fungicidal polymers filling the wood cell walls to extend the modification of acetylation on wood [46].

### 3.2. Other Chemical Modification

Yang et al. modified wood cell walls using dimethyldichlorosilane (DMDCS) to improve the hydrophobicity and mechanical properties [47]. The modification mechanism of DMDCS is similar to the acetylation treatment. The DMDCS impregnates into wood cell walls and replaces the original hydroxyl groups on the wood cell wall, giving strong hydrophobic properties. The nanoindentation technology shows that the nanomechanical properties of wood cell walls improve significantly, but the elastic modulus and hardness decrease slightly.

Another chemical modification method is based on the graft polymerization of cellulose, which creates wood polymer composites (WPCs) using a single grafting of vinyl on the molecular structure of celluloses. The modified wood has both the excellent properties of wood and monomeric polymers, such as good decay resistance and physical properties. However, many monomers used for this modification are difficult to penetrate wood cell walls [48]. To reduce the difficulty of modifiers penetrating cell walls, Wang et al. grafted polystyrene onto cell walls of poplar wood using free-radical copolymerization. They first swelled wood cell walls using methacryloyl chloride, then esterified with the hydroxyl groups on the cell walls, reducing the number of hydroxyl groups on the cell walls. Thereafter, they immersed and heated the polystyrene to copolymerize in situ with the previous methacrylic groups in the cell cavities. This method of increasing vinyl polymerization rate by substituting other components for hydroxyl groups successfully avoids the effect of wood cell wall permeability and also improves the performance of the synthesized WPCs [49].

Guo et al. found that the preparation of wood-polymer composites by polymerization of active monomers could not make full use of the properties of the polymer. They proposed a two-step method to improve the problem of poor interface compatibility of wood-polymer. They firstly immersed the water-soluble vinyl monomer into the cell walls, then reacted with the active double bond of the water-soluble vinyl monomer in the treated wood using styrene. The modified wood has a higher swelling resistance, hydrophobicity, and mechanical properties [50].

Ding studied the effect of composite modifier impregnation on the multi-function of wood. They prepared a multi-functional composite modifier using methyl urea, dimethyl dihydroxy ethylene urea (DMDHEU), urea, and silica sol as raw materials and treated wood. A cross-linking reaction occurs between wood cell walls and the composite modifier. Spectral analysis proves that this chemical modification has no significant effect on the crystal structure of cell wall cellulose, the hydroxyl groups are reduced, the thermal stability and mechanical properties of modified wood increase, and the moisture absorption decrease [51].

### 3.3. More on Modifications of Pores in Wood Cell Walls

Different from acetylation, part of the modification of wood is to modify the pores in wood cell walls [52]. Modifiers improve the properties of wood cells by filling and blocking micro-pores in the cell wall in the form of volume expansion. The cell wall pore sizes of wood are different with moisture content, thus resulting in different modification mechanisms and effects [53]. Using acetone as a solvent, He diffused maleic anhydride into the wood cell wall to expand the wood matrix, he reduced the hydroxyl groups, the water absorption capacity, the swelling of wood cell walls, and improved decay resistance of the wood cell walls [54]. In the studies of Noel et al. [55,56], the use of four biopolymers of polylactic acid, polyglycolic acid, aggregated butylene diacid, and polybutylene succinate were compared. Biopolymers have a good affinity with wood cell walls. During the heating and polymerization stage, they migrate to the cell walls, fill and expand in the cell walls, enhancing dimensional stability. However, biopolymers do not have good biological resistance.

The relevant modification methods also include furfurylation, thermosetting resin impregnation, etc. [57], and electron beam radiation (such as ultraviolet light) which is used to solidify substances that enter the cell walls [58]. Using tartaric acid as a catalyst, Hadi et al. impregnated furfuryl alcohol solution into cell walls of wood to improve the bio-resistance. The reagent accumulates in the cell walls and improves the hydrophobicity and decay resistance [59]. Liu et al. avoided the disadvantageous deposition of furfural resin in the cell cavity using vapor phase furfurylation and ensured the furfurylation of the cell wall. This technology promotes the further application of the furfurylation of wood [60]. Guo impregnated wood with the activated glucose group, activated glucose-based treatment agents can enter the micropores of the wood cell wall, produce a swelling effect on the wood, and improve the dimensional stability of wood. When the concentration of activated glucose was below 9%, the cross-linking between macromolecules in the cell wall was enhanced and the wet swelling rate of wood was reduced. When the concentration of activated glucose reached 18%, the cross-linking and damage to the cell wall reached an equilibrium [61].

Mahumut et al. used UV light to create a hydrogel by polymerizing polyethylene glycol diacrylate free radicals into the pores of the wood cell walls, and then modified wood using the hydrogel. The cross-links of polyethylene glycol diacrylate and hydrogels in the cell walls and cell cavities of wood were observed through confocal Raman microscopy, spectroscopy, and electron microscopy. This polymerization and cross-linking in the form of the hydrogel can react in water in a short time [62]. Wang also used free-radical copolymerization to graft polystyrene onto the cell wall of poplar wood. The graft copolymerization reaction improves the dimensional stability and surface hardness of the wood, as well as the hydrophobicity and wood-polymer interface compatibility [49]. Wang et al. explored the effect of polymeric diphenylmethane diisocyanate (pMDI) on the nano-mechanical properties of wood cell walls and detect the nano-chemical and nano-mechanical properties of wood cell walls using infrared radiation atomic force microscopy (AFM-IR) and nano-indentation technology. They concluded that pMDI can strengthen the original connection of cell wall polymer and improve wood elastic modulus and short-term creep resistance [63].

### 3.4. Flame Retardant Treatment

The commonly used flame retardants substances in wood and bamboo include inorganic salts, phosphoric acid, phosphate, ammonium sulfate, ammonium chloride, borax, boric acid, etc. [64]. The properties of flame retardants depend on the chemical structure of various retardants [65]. During the pyrolysis process, the phosphorus flame retardant is converted into phosphoric acid, which extracts water and carbon dioxide from the wood matrix during condensation, thereby isolating oxygen [66]. Sanjeet modified radiata pine using a two-step method of dimethyl methyl phosphate (DMMP). The result shows that the two-step method improves the thermal performance of flame retardants and the flame retardancy of wood [67]. The flame retardants containing phosphorus can remove the O and H atoms from the radical pool, thus reducing the hydroxyl groups indirectly [68] and carbonizing the wood, which may play as a further flame retardant. Lewin used bromide to modify wood and improved the flame resistance and decay resistance, while the mechanical property does not decline [69].

### 3.5. Chemical Mineralization of Wood

The mineralization process of the wood in nature takes millions of years, in which the silicic acid penetrates the cell walls and further transforms into a crystalline state through a polycondensation reaction [70]. Modern chemical methods can accelerate the process of the reaction and improve the properties of wood mineralization processing. Chemical mineralization usually uses organic or inorganic substances containing silicon. Wood is impregnated in the mineral solutions, and the mineral penetrates the cell wall and chemically reacts with some of its components, and remains in the cell cavities [71]. Doubek et al. used silica to modify wood in an attempt to affect its specific physical and mechanical properties as well as mold resistance. They impregnated beech and silver fir respectively with pressure using various concentration gradients of colloidal silica and then measured the physical and biological properties of the modified wood. The conclusion shows that silica improves the performance of beech and fir, but the anti-mold properties of wood are not ideal. The anti-mold properties did not change if the silica concentration is less than 10% [72]. Pressure treatment can accelerate the mineralization of wood, but silica does not react too much with the components of the cell walls, and only forms a slight covalent bond due to short time contact or big particle size of silica [73].

## 4. Other Innovative Modifications

Modern wood products are developing towards higher wood quality and performance. Multiple modification methods are developed to meet the needs of wood industries. The above-mentioned modification methods are also accompanied by other treatments in practical applications to finally obtain satisfactory modified wood. For the improvement of the cell wall, more research has focused on the multi-function, wide-areas, high-tech, and more comprehensive performance of wood.

### 4.1. Wood Carbonization Caused by Thermal Modification

Wood carbonization is associated with the TM technology of wood. Excessive temperature leads to the carbonization of wood components. The carbonized wood has lost a lot of its properties of wood, leaving more of the skeleton structure of wood cell walls. Xu studied some transformation characteristics of wood cell walls under carbonization using scanning thermal microscopy. This experimental method can effectively study the changes in the composition and structure of cell walls after carbonization, and provide ideas for the application of carbonized wood [74]. The development of wood carbonization has also gone through decades, mainly extending two research directions of wood ceramics and wood batteries. Wood ceramics retain the basic structural characteristics of wood cell walls, as well as being lightweight, having high specific strength, and a small expansion coefficient. Meanwhile, it also has good thermal conductivity, electrical conductivity, and wear resistance, having a wide range of applications [75]. Wood ceramics are prepared from wood powder, medium-density fiberboard, wood waste, and other wood materials, and modifiers are required to increase the performance of wood ceramics [76].

The wood energy storage materials are high-performance capacitors [77] which are constructed by the straight channels obtained by the carbonization of wood as a fast transmission channel for electrons and ions. Zhang et al. created an electrode using carbonized wood as a conductive frame by injecting Li metals. The channels formed on the cell wall structure of the carbonized wood are used as good guiding devices [78]. Chen et al. produced a high-performance ultra-thick electrode using carbonized wood as an energy storage matrix, then penetrated with lithium iron phosphate [79]. Zhang pointed out that wood-based electrodes have problems such as poor ion transmission capacity and poor mechanical properties of carbonized wood. Meanwhile, he indicated that future researches focus should be on the structural design of wood cell walls and other directions [80]. Suziki et al. firstly penetrated cobalt acetate into carbonized wood, then carbonized them again, and finally produced nanometer shell chains. This approach broadens the application of wood in the field of nano-carbons and provides a new direction to carbonized wood cell walls [81].

### 4.2. Aerogel-Based Woodmatrix Composites

Aerogel-based wood matrix composites are also named wood sponges. Wood has a natural porous cell structure, cellulose causes cell walls to be strong and tough, and lignin is the main component that causes wood discoloration and brittleness [82]. Moreover, through a series of subsequent functionalization (such as delignification, drying, functionalization), it could be endowed with a variety of new functions and then prepare biodegradable aerogel-type composite materials [83]. The aerogel-based wood matrix composites based on the functional modification to the structure and composition of the wood cell wall have advantages of low density, large specific surface area, high porosity, excellent mechanical properties, and environmental protection. This material has good application prospects in the fields of oil and water treatment, energy storage materials, and transparent materials [84]. Huang reported a wood sponge material with highly hydrophobic properties used for separating viscous crude oil and water, which are coated with reduced graphene oxide modified by fluoroalkylsilane [85]. Wang studied the effect of concentration of treatment reagents, pH, and other conditions on the wood sponge and optimized its performance of wood sponge. Under the conditions of pH 4.0 (NaCIO_2_ solution), NaOH (7 wt%), or NaCIO_2_ (3 wt%), wood sponges with a porosity of 96.47% were obtained, which has good resilience and excellent adsorption properties [86]. Mi et al. obtained an aerogel-type wood using delignified wood, and then impregnated polyvinyl alcohol to obtain a transparent wood with high light transmittance, thermal and mechanical properties [80].

### 4.3. Hydrogel-Based Woodmatrix Composites

Wang et al. prepared a composite hydrogel based on wood-matrix using delignified wood, which shows good performance in strength and swelling properties. He also delignifies balsa wood using sodium hypochlorite to obtain a transparent structure with a highly arranged nano-cellulose in the cell walls, then penetrates modified gelatin into wood cell walls to completely glue and cross-link the composite material [87]. Their work demonstrated wood structure is an excellent template for the preparation of high-performance composite hydrogels. The prepared hydrogel composite material based on the structure and composition of the cell wall presents new properties and could be a new idea and technology being widely used in the future.

### 4.4. Transparent Wood

Recently, some studies focus on the transparent wood [88,89,90]. The application of transparent wood mainly depends on its light transmission properties. The optical properties of transparent wood are also anisotropic due to the special structure of wood cell walls [91,92]. Chen studied the difference in light transmittance of acetylated and non-acetylated transparent wood with different thicknesses. He found the attenuation relationship between the transmittance and the thickness using the photon diffusion equation. The differences in the refractive index of the wood cell wall and the interface between the modifier and air cause the light transmittance of transparent wood to decrease. Acetylation improves the interface relationship and increases the light transmittance of wood [93].

### 4.5. Acoustic Performance

Wood has special natural acoustic characteristics due to its unique structure and thus it is used in various musical instruments. Despite the excellent sound performance, wood also has great sound absorption capacity and is used for noise control. Kang et al. changed the original porosity of wood through the steam explosion and increased the sorption coefficient of wood by 79%. Wang et al. increased the permeability of the pores on the cell wall using microwave treatment, and significantly enhanced the mid-frequency sound absorption performance of the modified wood. When microwave intensity, the thickness of the wood, and processing time were controlled at 18 kW, 30 mm, and 80 s, respectively, the optimal sound absorption was achieved for the treated wood [94]. TM at higher temperatures than a microwave, processes also improve the sound absorption performance of Specific wood. The increased permeability in the longitudinal of *Homalium foetidum* TM at 190 °C results in enhanced sound absorption [95].

## 5. Nanotechnology

A very important step in cell wall modification is to allow the modifier to penetrate cell walls or cell cavities before reacting. The channels through which the modifiers penetrate the cell walls are the pores and cavities on the cell walls. These pores are composed of pits and gaps among microfibrils [96]. These pores are all nanometers in diameter [97]. Research on the nano-pores of wood cell walls is of great significance to the modification of cell walls. Regardless of the modification method, the pore structures of wood cell walls are needed for further treatments.

In the application of nanotechnology modification, nanoparticles can penetrate the cell wall through certain means, but there are still technical difficulties. If the modifier does not penetrate the cell wall, the modifications to the wood are weak [98]. Mahr prepared new wood inorganic composites using the sol-gel method, and the nanometer titanium dioxide was uniformly generated in the wood cell wall, resulting in wood having good thermal and flame retardant properties [99]. Low-molecular-weight substances are easier to penetrate the wood cell walls, but high-molecular-weight substances usually only enter the wood cell cavities, resulting in weak modification [100,101,102]. Common wood modification focuses on creating wood more inert, while nanotechnology wood modification focuses on adding more functional properties to wood [103]. The most commonly used modifiers in nanotechnology are nano-titanium dioxide, nano-TiO_2,_ and ZnO, and they all can improve wood decay resistance [104] and flame retardant properties. Wood surfaces are modified after impregnating nano-clay, which enhances the mechanical, flame-retardant, and thermal stability of wood [105]. The sodium silicate was impregnated into the wood, forming a glassy state and a solid gel state in the wood cell walls and cavities, thus improving the overall hardness and flammability of the modified wood [106]. TiO_2_ was absorbed on the surfaces of wood through dip coating and annealing treatment, and the antibacterial properties, ultraviolet resistance, and a certain degree of self-cleaning ability of wood were improved [107]. The structure and composition of the cell wall are critical to the use of nanoparticle modifiers. The cell walls are needed to modify for the method of impregnation or coating to promote the nanoparticles entering the best position and exert the best performance.

## 6. Conclusions and Prospects

Wood cell walls play a key role in their physical, chemical, and mechanical properties. Modifications are applied to wood using various physical, chemical, and biological approaches to modify wood cell walls and improve additional functions and properties in previous studies and industry applications. Current modifications have been studied in new fields and ideas along with the further development of nanotechnology and microscopic observation technology. In this article, we list some interesting things and summarize the different modification methods. Some of them are creative, and because of their good performance, we think the modifications of wood and wood-based composites in the future should focus on three prospects: innovation of modifiers and combination with modification mechanism; wood cell walls, such as permeability, pore size and structures of wood cell walls; and application of nano-technologies.

(1) The research on traditional modification of TM, acetylation, and furfuralization should pay more attrition to the modifier innovation and combination of modifier and modification mechanisms. The permeability of wood is critical for modifiers penetrating; therefore, novel permeability improvement theory and technology should be developed in future research.
(2) Wood sponge, transparent wood, and energy storage materials are developed based on the modification to the wood cell wall and its structures. Future work should focus on the directional modification to the cell walls due to the particular anisotropic characteristic, the longitudinal, tangential, and radial mechanical properties of the cell wall can be individually enhanced to meet some special applications.
(3) Nanotechnology has been used in wood modification and provides more ideas for cell wall modification. Nanotechnology can not only be applied to modifiers to provide new ideas for solving the problem of cell wall permeability, but it can also be applied to cell wall structures. It is of great significance to study the cross-linking process of modifiers in cell walls from the nanoscale. The modification mechanism of nano-modifier particles, the distribution within the cell wall, and the nanostructure of the cell wall should be the key directions in future research.

## Data Availability

Not applicable.
